# The Impact of Heavy Load Carrying on Musculoskeletal Pain and Disability Among Women in Shinyanga Region, Tanzania

**DOI:** 10.5334/aogh.2470

**Published:** 2020-02-21

**Authors:** Jillian L. Kadota, Sandra I. McCoy, Michael N. Bates, Agatha Mnyippembe, Prosper F. Njau, Ndola Prata, Carisa Harris-Adamson

**Affiliations:** 1 Division of Epidemiology, School of Public Health, University of California, Berkeley, US; 2 Division of Environmental Health Sciences, School of Public Health, University of California, Berkeley, US; 3 Health for a Prosperous Nation, Dar es Salaam, TZ; 4 Prevention of Mother-to-Child HIV Transmission Programme, Ministry of Health, Community Development, Gender, Elderly, and Children, Dar es Salaam, TZ; 5 Bixby Center for Population and Sustainability, School of Public Health, University of California, Berkeley, US; 6 Department of Medicine, University of California, San Francisco, US

## Abstract

**Background:**

Heavy load carrying has been associated with musculoskeletal discomfort (MSD) and disability. However, there is a lack of research investigating this association in resource-constrained settings where heavy load carrying by women is common.

**Objective(s):**

We assessed the impact of heavy load carrying on musculoskeletal pain and disability among women in Shinyanga Region, Tanzania, in an exploratory cross-sectional study.

**Methods:**

Eligible participants were a convenience sample of women, at least 18 years of age, who passed a study recruitment site carrying a load. We collected information on load-carrying practices, including frequency and time spent carrying water, wood, agricultural products, coal, sand, or rocks, and measured the weight of the load carried at the time. Outcomes included self-reported MSDs, defined as experiencing pain lasting >3 days in the neck, head, back, knees, feet and/or ankles within the last 1 year, and related disability. Using multivariable logistic regression we assessed for associations between load carrying exposures and MSDs and disability.

**Findings:**

Results showed a high prevalence of MSDs across the body regions assessed and evidence to suggest a relationship of back pain and related disability with several measures of load-carrying, including duration, frequency, and weight. Multivariable analyses revealed associations of increased load carrying exposures with low back pain (LBP) and related disability, including statistically significant increases in odds of LBP with increasing weight, total duration of load carrying/week and cumulative loads/week.

**Conclusions:**

Findings indicate a substantial burden of MSDs and disability in this population of women who carry heavy loads daily. The extent of discomfort and disability increased with increasing exposure to various load-carrying measures, especially for LBP. Larger epidemiologic studies that definitively assess relationships of load carrying with MSDs and disability are warranted.

## Introduction

Musculoskeletal injuries comprise as the largest burden of disease globally. The 1990, 2005, and 2015 Global Burden of Disease studies listed low back pain (LBP) and neck pain as the primary global contributors to years lost to disability (YLD), and in the top ten causes of YLDs in all countries [1,2]. Musculoskeletal injuries like LBP can affect an individual’s capacity to carry out activities for daily living, including the ability to work and care for children. In low- and middle-income countries (LMIC) this can have serious implications for livelihoods and household welfare. As such, it is surprising that there has been relatively little research in LMIC to identify and mitigate the causes of musculoskeletal pain and disorders [3].

One possible cause of musculoskeletal discomfort (MSD) and eventual disorders is heavy load carrying, a common practice in many parts of the developing world where roads are poor or non-existent, automotive transportation is scarce or unaffordable, and household necessities, such as water, food and firewood, must be manually carried long distances. For example, in Nepal, a circular band called a ‘namlo’ is strapped around the forehead and used to carry loads, typically in a ‘doko’, a basket that often holds loads around 20–35 kg and sometimes much more [4,5]. In sub-Saharan Africa (SSA), load carrying is typically done with buckets or baskets balanced atop the head, commonly with loads of 25–35 kg, but up to 60 kg reported [3]. In many LMIC ‘domestic load-carrying’ is regarded as a low-status activity and culturally assigned to females; thus, women and girls bear much of the carrying burden [3].

The scant published research on load carrying in LMIC suggests that it negatively impacts musculoskeletal pain and function, especially for women and girls [3,6,7,8]. However, this evidence is mostly anecdotal or speculative [3]. Indeed, despite the wide prevalence of load-carrying activities amongst women and girls in LMIC there has been little investigation among these populations of its potential detrimental impacts [7]. Much of the existing research on this topic has been conducted in high-income countries or has largely focused on the experience of pain in adult male workers [9,10,11,12]. This evidence is not likely to be generalizable to women and girls who have different tissue capacities and injury tolerance associated with load-carrying activities than men [6]. Although one recent cross-sectional study conducted in South Africa, Ghana and Vietnam found evidence for associations between water carrying and upper back pain, assessments did not consider other loading materials, such as firewood, agricultural products, or sand/rocks [7].

Overall, this points to a need for more research assessing the biomechanical (degenerative changes and risk of acute injury), physiological (maternal and cardiovascular health) and psycho-social (pain and disability) impacts among women who carry different types of heavy loads in LMIC, often from a very young age [3]. Addressing this largely neglected topic, which may be responsible for a substantial burden of disease and disability worldwide, will begin to fill a significant knowledge gap and, if associations with load carrying are found, will encourage further studies of this relationship. If confirmed, the development and implementation of interventions and/or policy recommendations should be considered to reduce harm, pain, and loss of function among women and girls in LMIC. The aim of this study was to help fill the data gaps by collecting preliminary data on potential associations between heavy load carrying and musculoskeletal discomfort and disability among women in Tanzania. As information on this issue is scarce, we sought to cast a wide net to find associations that might generate hypotheses that could be followed up with more targeted future research.

## Methods

The institutional review board of the University of California, Berkeley and the National Institute of Medical Research (NIMR) of Tanzania approved the study protocol before data collection began.

### Study participants

A cross-sectional study was conducted amongst women who carry loads as part of their activities for daily living (collecting water, firewood, etc.), recruited from 6 different study sites in Shinyanga Region, Tanzania. This region, which has a population of 1.6 million, is one of the 26 regions of mainland Tanzania and is located in the Lake Zone. Participant recruitment sites were established at a convenience sample of locations near water collection points and along farm and market routes; each site was chosen based on local knowledge, accessibility by foot or by automobile, and consideration of the amount of foot traffic in the area during daylight hours. Eligible participants were a convenience sample of women of at least 18 years of age who passed a study recruitment site carrying a load and were able and willing to participate. Before participation, consent forms were read to participants who provided either a signature or a thumbprint indicating their consent. Although most people living in Shinyanga speak Sukuma, it is not traditionally a written language. Because of this, and because generally the local population could also speak and/or read Swahili, interviews were administered in Swahili by a trained interviewer who was a member of the study team. Subjects who did not speak or understand Swahili were excluded.

### Data collection

The primary data collection instrument was a structured survey. In developing the survey, the main constructs were informed by expert local knowledge, observations of study locations, types of loads frequently carried there by women, and existing literature on load carrying and related health impacts. Information collected included social and community factors and individual characteristics. The survey was comprised of the following sections: 1) socio-demographics, 2) load-carrying practices, 3) overall health/reproductive health, 4) musculoskeletal pain and 5) disability due to musculoskeletal pain. The interview took approximately 30 minutes. Participants’ height (centimeters) and weight (kilograms) were measured and used to calculate the body mass index (BMI) (kg/m^2^). Primary data collection took place across 10 days in July and August of 2016.

### Load-carrying exposures

Participants were asked about experiences carrying loads of water, wood, agricultural products (grains, corn, ground corn, etc.), coal, sand, and rocks. Questions asked about the types of loads carried ever and within the last 7 days, the frequency of load carrying, the average time and distance travelled while carrying loads in the last 7 days, and the age that the participant first engaged in load carrying. The Borg CR-10 rating scale was used to assess the perceived difficulty in carrying loads; it has previously been used in similar populations [7,13]. Participants were asked to quantify perceived difficulty of carrying various types of loads on a 0–10 rating scale, with 0 being ‘no difficulty at all’, 1 being ‘very slight’, 2 being ‘fairly slight’, 3 being ‘moderate’, 4 being ‘somewhat hard’, 5 being ‘hard’, 7 being ‘very hard’, and 10 being ‘very, very hard’ (maximal). Additionally, the type of load carried on the interview date, method of load carriage (i.e., on head, on back, in hand, etc.), footwear, and additional loads carried by the participant at the time of the interview, such as a baby, were noted by the interviewer. Force gauges and scales were used to measure the weight of the load carried on the day of the interview.

For validation purposes, we collected corroborating evidence for the distance and time spent carrying loads from a sub-sample of the study participants using a wristwatch-like device enabled with GPS capabilities. Using this device, we assessed distance traveled by women over a 24-hour wearing period. Participants were instructed to record each time a new load was carried by pressing a button on the device. Their recall of load-carrying activities was also assessed in a survey directly following the 24-hour wearing period.

### Outcomes

The primary outcomes included self-reported musculoskeletal pain, defined in the survey as experiencing pain lasting more than 3 days – in the neck, head, back, knees, feet and/or ankles – within the last 1 year. Amongst those with >3 days of pain, information on the pain frequency (number of days in the last month) and severity (0–10 NRS) was assessed by body region [14]. The occurrence of disability was assessed by interview and served as a secondary outcome. This included the impact of musculoskeletal pain over the prior year on activities for daily living, such as: 1) cooking, 2) caring for others, such as children or elderly, 3) taking care of the home, or 4) collecting wood, water, or other household necessities. We also assessed the number of missed workdays within the last month due to the body region-specific musculoskeletal pain. Finally, participants were asked to identify other types of activities, if any, they found difficult to complete because of musculoskeletal pain.

### Statistical analysis

#### Covariates

Age, BMI (normal/underweight vs. overweight), marital status (unmarried/single vs. married/with partner), overall health rating (good/very good/excellent vs. poor/fair), parity, and age at first child were examined for associations with the outcome variables using unadjusted bivariate logistic regression and were considered as potential covariates in the primary exposure-outcome models. The categories for the above-assessed covariates were determined according to established cut points previously used in the literature and, when necessary, by combining groups to ensure adequate sample size in each category.

#### Multivariable modeling

Multivariable models were constructed to assess the relationship between various measures of load carrying and the odds of musculoskeletal pain across body regions. Specific exposures assessed in our multivariable models included the measured weight of the load carried on the interview date (kilograms), the average duration of load carrying per trip within the last week (minutes), and the number of loads carried per week, both self-reported, and a cumulative estimate calculated by multiplying the average number of loads carried per day by the number of days carrying loads within the last week. To assess the combined factors of load weight, duration, and frequency, two composite exposure measures were also calculated: total duration of any load carrying per week (duration*number of loads carried/week) and cumulative load weight carried per week (measured weight of load in kilograms*average duration in minutes*number of loads carried/week). Each exposure variable was divided into low, medium, and high exposure tertiles. The occurrences of musculoskeletal pain in each body region (head, neck, back, knee, and/or feet/ankle pain) lasting more than 3 days over the last 12 months served as our primary outcomes and were assessed as binary variables. We also estimated the odds of musculoskeletal pain with weight of load (kilograms) and load-carrying duration (minutes) modeled as continuous variables.

Each load-carrying exposure and body region-specific musculoskeletal pain relationship was modeled separately. Models were constructed using a generalized linear structure with a logit link to estimate odds ratios and corresponding 95% confidence intervals (95% CIs), and were adjusted for covariates found to be associated with outcome variables in our bivariate analyses and/or were factors included as potential confounders in similar previous studies, including age, parity, and BMI [15,16,17,18,19,20]. We assumed the study population median age for participants with unknown age (n = 9). Similarly, we constructed body region-specific multivariable logistic regression models in order to assess the impact of various load-carrying exposures on our secondary outcome of interest – experiencing disability due to musculoskeletal pain.

## Results

Out of the 132 individuals approached, 95% (n = 125) were eligible. Of these, 65.6% (n = 82) consented to participate in the study. The primary reason given for non-participation was lack of time.

### Sample characteristics

Participating women had a mean age of 31.6 years (SD: 12.2); although all participants confirmed being over the age of 18, nine reported not knowing their specific age. Most participants indicated their occupation as farming (95%) and were either married or living with a partner (74.4%). The average household size was 7.5 people (SD: 3.8), and 77 (93.9%) had children. The average number of children per participant was approximately 4 (Table 1).

**Table 1:** Characteristics of the study sample of women, Shinyanga Region, Tanzania, July–August 2016.

Participant Characteristic	Mean (SD)	N (%)

**Age (years)**	31.6 (12.2)	
18–30		47 (57.3)
>30		26 (31.7)
Unknown age		9 (11.0)
**BMI (kg/m^2^)**	24.4 (4.2)	
Underweight (<18.5)		2 (2.4)
Normal (18.5–25)		55 (67.1)
Overweight (>25)		27 (32.9)
**Marital status**		
Married/with partner		61 (74.4)
Unmarried/Single		21 (25.6)
**Occupation**		
Farmer		78 (95.1)
Other		4 (4.9)
**Primary Language**		
Swahili		3 (3.7)
Sukuma		77 (93.9)
Other		2 (2.4)
**Overall Personal Health Rating**		
Poor/Fair		50 (61.0)
Good/Very Good/Excellent		32 (39.0)
**Parity**	4.0 (2.5)	
0–3 children		45 (54.9)
≥4 children		37 (45.1)
**Number of lifetime pregnancies**	4.4 (3.1)	
None		3 (3.7)
1–2 pregnancies		13 (15.9)
3–4 pregnancies		33 (40.2)
≥5 pregnancies		33 (40.2)
**Age at first child (n = 66)**	18.1 (2.2)	
13–19 years		42 (63.6)
≥19 years		24 (36.4)

**BMI** = body mass index.

### Summary of load-carrying exposures

Within the prior week, 94% (n = 77) of the women had carried water, 84% (n = 69) had carried agricultural products, and 70% (n = 57) had carried wood (Table 2). Half (50%) of all study participants had ever carried sand, while approximately 40% had ever carried rocks or coal. The weight, frequency and duration of the load carried varied by the material. For example, on average among wood carriers, wood was collected 1.4 times per day (SD: 0.8), 2.4 days per week (SD: 1.6), with an average trip time of 81.2 minutes (SD: 53.1), while water, with an average trip time of 18.8 minutes (SD: 12.3) was collected on average 5.3 days per week (SD: 2.0), 4.0 times per day (SD: 2.0) (Table 2). Information collected using GPS devices and follow-up surveys from a sub-sample of study participants (n = 14) corroborates these self-reported data, revealing an average of 3.64 load-carrying trips per day (range: 1–9 trips) covering an average distance of 5.32 miles over the course of the 24-hour wearing period.

**Table 2:** Summary of load carrying exposure measures, Shinyanga Region, Tanzania, July–August 2016.

Type of load	Ever Carried	Carried item last 7 days^1^	Days carrying loads last 7 days	Number of loads per day last 7 days	Minutes per trip last 7 days	Borg CR-10 rating

	N (%)	N (%)	Mean (SD)	Mean (SD)	Mean (SD)	Mean (SD)

Any Load	82 (100)	—	5.3 (1.8)	3.7 (2.2)	29.5 (18.8)	—
Water	82 (100)	77 (93.9)	5.3 (2.0)	4.0 (2.0)	18.8 (12.3)	4.0 (1.6)
Wood	82 (100)	57 (69.5)	2.4 (1.6)	1.4 (0.82)	81.2 (53.1)	5.7 (1.7)
Agricultural products	82 (100)	69 (84.2)	2.8 (1.8)	1.8 (1.0)	48.2 (33.4)	5.2 (2.0)
Coal	31 (37.8)	7 (22.6)	1.7 (0.76)	1.3 (0.49)	78.6 (53.0)	3.2 (1.6)
Sand	41 (50.0)	6 (14.6)	2.2 (1.5)	3.8 (1.2)	18.7 (20.7)	6.7 (1.7)
Rocks	33 (40.2)	3 (9.1)	2.0 (1.7)	3.7 (1.2)	64 (100.5)	6.9 (1.8)
Bricks	17 (20.7)	17 (100)	2.2 (1.1)	11.8 (4.7)	12.8 (13.3)	6.0 (2.4)

^1^ Percentage of those who indicated having ever carried.

On the interview date, women were carrying water (n = 51; 62.2%), wood (n = 17; 20.7%), and agricultural products (n = 14; 17.1%). The average weight of all loads measured (n = 80) was 18.8 kg (SD: 5.8): water weighed an average of 18.9 kg (SD: 4.4), wood 20.9 kg (SD: 9.3), and agricultural products 16.2 kg (SD: 4.6). All participants carried their load atop their head, and almost all wore plastic, open-toed shoes. Of the 82 participants, 16 (19.5%) were carrying an additional load: 15 women were carrying a baby on their back, and 1 participant was carrying a bucket of ground maize in her hand.

Using the Borg CR-10 scale to quantify perceived difficulty of carrying various types of loads, rocks (\;\bar X = 6.9,\;SD:1.8), sand (\bar X = 6.7,\;SD:1.7), and bricks (\overline X = 6.0,\;SD:2.4) were rated the most difficult (Table 2). Among the products carried, water was rated on average as ‘Somewhat hard’ (\;\bar X = 4.0,\;SD:1.6), and wood and agricultural products were rated between ‘Hard’ and ‘Very hard’ (wood: \bar X = 5.7,\;SD:1.7; agricultural products: \bar X = 5.2,\,\;SD:2.0).

### Prevalence of MSDs and disability

Over two-thirds of participants reported experiencing neck pain lasting more than three days within the last year and 40–50% of participants reported head, back, knee, or feet/ankle pain lasting more than three days within the last year (Table 3). On average, participants rated pain in all parts of the body as being slightly above ‘Moderate’, or approximately equivalent to 6 on a 0–10 pain rating scale [14].

**Table 3:** Summary of musculoskeletal discomfort and disability outcome measures, Shinyanga Region, Tanzania, July–August 2016.

Musculoskeletal Discomfort	Prevalence			Severity		Tasks difficult to complete among those with musculoskeletal pain during last year)				

	Pain >3 days in last 1 year	Pain >3 days in last 1 month	Number of days of pain, last 1 month	Number of days unable to work last 1 month due to pain	Pain Rating (0-10 scale)^1^	Cooking	Taking care of the home	Caring for others (children, elderly)	Collecting wood, water, other necessities	Farming^2^

	N (%)	N (%)	Mean (SD)	Mean (SD)	Mean (SD)	N (%)	N (%)	N (%)	N (%)	N (%)

Neck	50 (61.0)	25 (30.5)	6.6 (6.2)	2.1 (2.7)	6.3 (2.1)	24 (48.0)	23 (46.0)	19 (38.0)	37 (74.0)	6 (12.0)
Head	35 (42.7)	18 (22.0)	6.3 (6.8)	2.4 (2.9)	6.1 (2.1)	19 (54.3)	21 (60.0)	17 (48.6)	26 (74.3)	3 (8.6)
Back	30 (48.8)	18 (22.0)	12.1 (11.3)	2.7 (2.6)	6.5 (2.2)	15 (50.0)	14 (46.7)	12 (40.0)	27 (90.0)	0 (0)
Knees	35 (42.7)	22 (26.8)	15.1 (12.1)	1.2 (1.7)	5.8 (2.3)	11 (31.4)	10 (28.6)	9 (25.7)	22 (62.9)	2 (5.7)
Feet/Ankles	39 (47.6)	29 (35.4)	14.0 (12.3)	1.3 (2.2)	6.1 (2.6)	8 (20.5)	10 (25.6)	8 (20.5)	20 (51.2)	3 (7.7)

^1^ Scale was on a 0–10 rating, with 0 being ‘No pain’, 5 being ‘Moderate pain’, and 10 being ‘Worst Pain’. ^2^ Self-identified. **MSD** = musculoskeletal discomfort.

Disability, defined as having difficulty completing activities for daily living due to musculoskeletal pain within the last year, was experienced across musculoskeletal regions. In addition to the activities identified *a priori* (cooking, caring for the home, caring for others, and collecting wood, water or other necessities), some participants reported having difficulty farming. The most prevalent activity affected by pain was collecting wood, water, or other household necessities, with 51–90% of participants reporting such disability (Table 3).

### Results from multivariable modeling of MSDs and related disability

**Figure 1:**
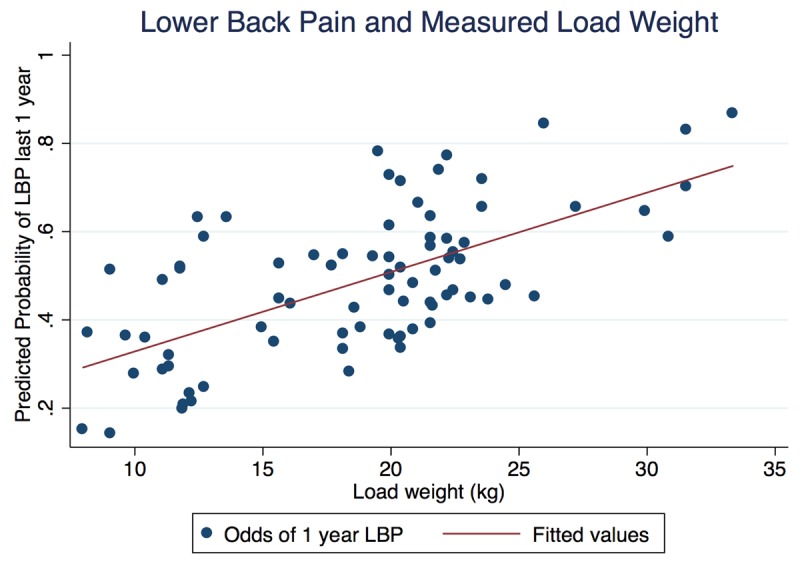
Predicted probability of low back pain with measured load weight in kilograms (continuous), Tanzania, July–August 2016.

Overall, our primary multivariable analyses revealed consistent associations between increasing load-carrying exposures and greater odds of low back pain, and some evidence for increased odds of knee pain. Generally, odds of pain became greater as loads became heavier or trip durations became longer. For example, 32.6% (95% CI: 13.7–51.6) of those in the low load weight category, 50.6% (95% CI: 31.6–69.7) in the medium category, and 64.8% (95% CI: 44.8–84.7) of those in the high load weight category experienced LBP lasting more than 3 days within the last year (results not shown). Using the best-fitting line for our regression with load weight modeled as a continuous variable, a woman carrying the average load weight of 18.8 kg had an approximately 50% predicted probability of low back pain within the last year (Figure 1). There was a statistically significant increase in odds of LBP associated with increasing weight of load, total duration of load carrying/week, and cumulative loads/week (Table 4). Models using measured load weight and load-carrying duration as continuous exposure variables provided evidence of significantly increased odds of LBP, with a 10% increase in odds for each one-kilogram increase in load weight. We also found 2.2 times the odds for every 30-minute increase in load-carrying duration. Generally, the odds of knee pain increased with increasing load-carrying exposures. The odds of experiencing head pain also increased with greater average number of loads per week, although the confidence interval included the null value. Overall, we found no evidence for increased odds of neck or foot/ankle pain with increasing load-carrying exposure. To confirm our results, we conducted post-hoc analyses including an assessment of severe pain (pain rating >5 on a 10-point scale) within the last 12 months, as well as odds of pain for every unit increase in load weight within the last month. We found our results to be consistent with our 12-month primary outcome models, including increased odds of severe back pain with increasing exposure to various load carrying exposures, and increased odds of LBP within the last month with each 1 kg increase in load weight (aOR = 1.14, 95% CI: 1.02–1.28).

**Table 4:** Adjusted odds ratios (ORs)^1^ for reported musculoskeletal discomfort within the last year according to various measures of load carrying exposure, Shinyanga Region, Tanzania, July–August 2016.

Characteristics	Musculoskeletal Discomfort																			

	Head pain lasting >3 days, last 12 months				Neck pain lasting >3 days, last 12 months				Back pain lasting >3 days, last 12 months				Knee pain lasting >3 days, last 12 months				Feet/Ankle pain lasting >3 days, last 12 months			

	No (n = 47)	Yes (n = 35)	OR	95% CI	No (n = 32)	Yes (n = 50)	OR	95% CI	No (n = 42)	Yes (n = 40)	OR	95% CI	No (n = 47)	Yes (n = 35)	OR	95% CI	No (n = 43)	Yes (n = 39)	OR	95% CI

**Weight of load (kg) (n = 80)**																				
Low	14 (29.8)	13 (39.4)	1.00		11 (35.5)	16 (32.7)	1.00		17 (41.5)	10 (25.6)	1.00		17 (37.0)	10 (29.4)	1.00		14 (33.3)	13 (34.2)	1.00	
Medium	22 (46.8)	7 (21.2)	0.29	(0.09, 0.97)	12 (38.7)	17 (34.7)	0.91	(0.29, 2.80)	15 (36.6)	14 (35.9)	2.12	(0.62, 6.86)	14 (30.4)	15 (44.1)	2.44	(0.71, 8.39)	18 (42.9)	11 (29.0)	0.58	(0.18, 1.88)
High	11 (23.4)	13 (39.4)	1.13	(0.34, 3.67)	8 (25.8)	16 (32.7)	1.19	(0.35, 4.02)	9 (22.0)	15 (38.5)	3.79	(1.07, 13.47)	15 (32.6)	9 (26.5)	1.23	(0.34, 4.55)	10 (23.8)	14 (36.8)	1.30	(0.39, 4.32)
**Average load carry duration (mins) (n = 82)**																				
Low	19 (40.4)	16 (45.7)	1.00		12 (37.5)	23 (46.0)	1.00		23 (54.8)	12 (30.0)	1.00		19 (40.3)	16 (45.7)	1.00		18 (41.9)	17 (43.6)	1.00	
Medium	18 (38.3)	14 (40.0)	0.86	(0.32, 2.33)	13 (40.6)	19 (38.0)	0.71	(0.25, 1.98)	14 (33.3)	18 (45.0)	2.12	(0.77, 5.87)	20 (42.6)	12 (34.3)	0.45	(0.17, 1.39)	20 (46.5)	12 (30.8)	0.46	(0.16, 1.35)
High	10 (21.3)	5 (14.3)	0.52	(0.14, 1.90)	7 (21.9)	8 (16.0)	0.55	(0.16, 1.96)	5 (11.9)	10 (25.0)	3.46	(0.93, 12.80)	8 (17.0)	7 (20.0)	0.73	(0.19, 2.77)	5 (11.6)	10 (25.6)	1.76	(0.47, 6.60)
**# of loads/Week (n = 82)**																				
Low	19 (40.4)	9 (25.7)	1.00		11 (34.4)	17 (34.0)	1.00		15 (35.7)	13 (32.5)	1.00		19 (40.4)	9 (25.7)	1.00		11 (34.4)	17 (34.0)	1.00	
Medium	19 (40.4)	14 40.0)	1.74	(0.59, 5.16)	14 (43.8)	19 (38.0)	0.78	(0.27, 2.24)	17 (40.5)	16 (40.0)	1.09	(0.38, 3.15)	19 (40.4)	14 (40.0)	1.74	(0.59, 5.16)	14 (43.8)	19 (38.0)	0.78	(0.27, 2.24)
High	9 (19.2)	12 (34.3)	3.43	(0.97, 12.02)	7 (21.9)	14 (28.0)	1.00	(0.29, 3.53)	10 (23.8)	11 (27.5)	1.27	(0.37, 4.31)	9 (19.2)	12 (34.3)	3.42	(0.97, 12.02)	7 (21.9)	14 (28.0)	1.00	(0.29, 3.53)
**Total Duration/Week (mins) (n = 82)**																				
Low	17 (36.2)	12 (34.3)	1.00		11 (34.4)	18 (36.0)	1.00		18 (42.9)	11 (27.5)	1.00		17 (36.2)	12 (34.3)	1.00		15 (34.9)	14 (5.9)	1.00	
Medium	16 (34.0)	11 (31.4)	1.14	(0.37, 3.54)	12 (37.5)	15 (30.0)	0.57	(0.18, 1.82)	16 (38.1)	11 (27.5)	1.14	(0.35, 3.72)	17 (36.2)	10 (28.6)	1.20	(0.35, 4.08)	17 (39.5)	10 (25.6)	0.58	(0.18, 1.91)
High	14 (29.8)	12 (34.3)	1.12	(0.37, 3.35)	9 (28.1)	17 (34.0)	1.01	(0.32, 3.18)	8 (19.1)	18 (45.0)	3.70	(1.15, 11.89)	13 (27.7)	13 (37.1)	1.35	(0.41, 4.38)	11 (25.6)	15 (38.5)	1.19	(0.38, 3.68)
Cumulative load (kg*mins/Week) (n = 80)																				
Low	16 (34.0)	11 (33.3)	1.00		11 (35.5)	16 (32.7)	1.00		17 (41.5)	10 (25.6)	1.00		18 (39.1)	9 (26.5)	1.00		13 (31.0)	14 (6.8)	1.00	
Medium	17 (36.2)	10 (30.3)	0.88	(0.28, 2.74)	11 (35.5)	16 (32.7)	0.88	(0.28, 2.74)	16 (39.0)	11 (28.2)	1.47	(0.46, 4.73)	15 (32.6)	12 (35.3)	2.79	(0.77, 10.15)	17 (40.5)	10 (26.3)	0.54	(0.17, 1.72)
High	14 (29.8)	12 (36.4)	1.16	(0.38, 3.59)	9 (29.0)	17 (34.7)	1.14	(0.36, 3.65)	8 (19.5)	18 (46.2)	3.94	(1.19, 13.07)	13 (28.3)	13 (38.2)	2.16	(0.61, 7.69)	12 (28.6)	14 (36.8)	0.83	(0.26, 2.60)

^1^ Adjusted for age, BMI, and parity.

Our secondary multivariable analysis assessing the impact on activities for daily living revealed similar trends to our primary analysis. Although confidence intervals included the null, the odds of disability due to back pain increased with increasing exposure to load weight, average carrying duration, and number of loads per week. There was a significant increase in odds of disability due to knee pain with increasing number of loads per week and cumulative load (Table 5).

**Table 5:** Odds ratios (ORs) for reported disability due to musculoskeletal pain within the last year according to various measures of load carrying exposure, Shinyanga Region, Tanzania, July–August 2016^1^.

Characteristics	Disability due to pain																			

	Disability due to head pain, last 12 months				Disability due to neck pain, last 12 months				Disability due to back pain, last 12 months				Disability due to knee pain, last 12 months				Disability due to feet/ankle pain, last 12 months			

	No (n = 55)	Yes (n = 27)	OR	95% CI	No (n = 43)	Yes (n = 39)	OR	95% CI	No (n = 54)	Yes (n = 28)	OR	95% CI	No (n = 60)	Yes (n = 22)	OR	95% CI	No (n = 60)	Yes (n = 20)	OR	95% CI

**Weight of load (n = 80)**																				
Low	15 (27.3)	12 (48.0)	1.00		13 (31.0)	14 (36.8)	1.00		19 (35.9)	8 (29.6)	1.00		20 (33.9)	7 (33.3)	1.00		21 (34.4)	6 (31.6)	1.00	
Medium	23 (41.8)	6 (24.0)	0.29	(0.08, 1.04)	18 (42.9)	11 (29.0)	0.54	(0.18, 1.64)	20 (37.7)	9 (33.3)	1.09	(0.32, 3.77)	21 (35.6)	8 (38.1)	1.19	(0.33, 4.37)	22 (36.1)	7 (36.8)	1.19	(0.30, 4.67)
High	17 (30.9)	7 (28.0)	0.47	(0.14, 1.64)	11 (26.2)	13 (34.2)	1.02	(0.32, 3.25)	14 (26.4)	10 (37.0)	1.66	(0.47, 5.79)	18 (30.5)	6 (28.6)	0.99	(0.25, 3.87)	18 (29.5)	6 (31.6)	1.12	(0.28, 4.56)
**Average load carry duration (n = 82)**																				
Low	24 (43.6)	11 (40.7)	1.00		9 (26.5)	13 (37.1)	1.00		15 (34.9)	7 (26.9)	1.00		15 (31.3)	7 (33.3)	1.00		15 (30.0)	7 (36.8)	1.00	
Medium	20 (36.4)	12 (44.4)	1.14	(0.40, 3.27)	16 (47.1)	16 (45.7)	1.02	(0.38, 2.72)	21 (48.8)	11 (42.3)	1.25	(0.41, 3.80)	23 (47.9)	9 (42.9)	1.03	(0.32, 3.32)	25 (50.0)	7 (36.8)	0.70	(0.20, 2.45)
High	11 (20.0)	4 (14.8)	0.66	(0.16, 2.67)	9 (26.5)	6 (17.1)	0.41	(0.19, 2.34)	7 (16.3)	8 (30.8)	2.92	(0.78, 11.01)	10 (20.8)	5 (23.8)	1.35	(0.33, 5.54)	10 (20.0)	5 (26.3)	0.74	(0.32, 5.76)
**# of Loads/Week (n = 82)**																				
Low	20 (36.4)	8 (29.6)	1.00		13 (30.2)	15 (38.5)	1.00		19 (35.2)	9 (32.1)	1.00		24 (40.0)	4 (18.2)	1.00		20 (32.3)	8 (40.0)	1.00	
Medium	22 (40.0)	11 (40.7)	1.37	(0.44, 4.23)	16 (37.2)	17 (43.6)	0.84	(0.30, 2.37)	22 (40.7)	11 (39.3)	1.06	(0.34, 3.32)	21 (35.0)	12 (54.6)	4.32	(1.09, 17.19)	27 (43.6)	6 (30.0)	0.49	(0.13, 1.83)
High	13 (23.6)	8 (29.6)	1.76	(0.49, 6.36)	14 (32.6)	7 (18.0)	0.36	(0.10, 1.24)	13 (24.1)	8 (28.6)	1.20	(0.33, 4.41)	15 (25.0)	6 (27.3)	2.82	(0.59, 13.38)	15 (24.2)	6 (30.0)	0.80	(0.20, 3.28)
**Total Duration/Week (mins) (n = 82)**																				
Low	19 (34.6)	10 (37.0)	1.00		13 (30.2)	16 (41.0)	1.00		21 (38.9)	8 (28.6)	1.00		24 (40.0)	5 (22.7)	1.00		21 (33.9)	8 (45.0)	1.00	
Medium	19 (34.6)	8 (29.6)	0.94	(0.28, 3.14)	16 (37.2)	11 (28.2)	0.48	(0.15, 1.49)	20 (37.0)	7 (25.0)	0.91	(0.25, 3.37)	19 (31.7)	8 (36.4)	1.06	(0.27, 4.19)	22 (35.5)	5 (25.0)	0.31	(0.07, 1.32)
High	17 (30.9)	9 (33.3)	0.92	(0.29, 2.90)	14 (32.6)	12 (30.8)	0.64	(0.21, 1.90)	13 (24.1)	13 (46.4)	2.36	(0.73, 7.68)	17 (28.3)	9 (40.9)	2.26	(0.55, 9.20)	19 (30.7)	7 (35.0)	0.68	(0.17, 2.79)
Cumulative load (kg*mins/Week) (n = 80)																				
Low	18 (32.7)	9 (36.0)	1.00		12 (28.6)	15 (39.5)	1.00		19 (35.9)	9 (29.6)	1.00		23 (39.0)	4 (19.1)	1.00		20 (32.8)	7 (36.8)	1.00	
Medium	20 (36.4)	7 (28.0)	0.81	(0.24, 2.77)	17 (40.5)	10 (26.3)	0.44	(0.14, 1.35)	21 (39.6)	7 (22.2)	0.74	(0.20, 2.67)	18 (30.5)	9 (42.9)	4.67	(1.04, 20.97)	21 (34.4)	6 (31.6)	0.95	(0.24, 3.68)
High	17 (30.9)	9 (36.0)	0.99	(0.30, 3.26)	13 (31.0)	13 (34.2)	0.75	(0.25, 2.29)	13 (24.5)	13 (48.3)	2.04	(0.63, 6.64)	18 (30.5)	8 (38.1)	2.52	(0.59, 10.83)	20 (32.8)	6 (31.6)	0.64	(0.17, 2.49)

^1^ Adjusted for age, BMI, and parity.

## Discussion

Load carrying is an almost-ubiquitous activity amongst women in African countries, especially in more rural regions of the African continent where reliable and cheap mechanized transportation is often scarce. Previous reports, although mostly speculative or anecdotal, have suggested that there could be significant musculoskeletal impacts associated with heavy load-carrying activities, particularly for women and girls [3]. The present study investigates this claim in Shinyanga Region, Tanzania. Findings from our analysis indicate a substantial burden of musculoskeletal pain and disability in women carrying heavy loads, usually daily. Generally, we found that the extent of discomfort and disability increased with increasing exposure to various load-carrying measures, especially for low back pain.

Considering that all women in this study population were observed carrying loads atop their head, it is surprising that in our primary multivariable analysis of pain within the last 12 months, we found no strong relationship between neck or head pain and load exposure. Instead, our main finding was a consistent relationship of low back pain with measures of load-carrying duration, frequency, and weight, with some point estimates showing three-fold or more odds of one-year pain prevalence. Similar to our primary analyses of musculoskeletal pain, our secondary assessment of impacts on activities for daily living suggested increased odds of disability due to knee pain with increasing number of loads carried per week and increasing cumulative load, and increased odds of disability due to back pain with increasing load weight, average carrying duration, and number of loads/week.

While study results suggest important potential relationships between MSDs and load carrying, some effect estimates had wide, imprecise confidence intervals. This is likely to be because our modest sample size limited statistical power to precisely measure associations. We were also limited to a one-time, cross-sectional survey, thus restricting our ability to draw any firm causal associations between load carrying and MSDs and related disability; ideally, future studies will be larger and longitudinal. Additionally, our findings could be confounded by other daily activities associated with load carrying for which information was not obtained. This could include sustained or repetitive forward bending while farming, cooking, or caring for children. It is also probable that the actual lifting and lowering technique of the objects carried was a source of musculoskeletal discomfort, particularly for the lower back. Although we observed the manner in which women lifted and lowered their carried items, we did not formally document these exposures. We suggest that future studies also attempt to record static and repetitive bending, as well as the technique used to lift and lower loads throughout a typical day. Some selection bias is possible since we included a sample of convenience; it is plausible that our sample did not represent the population of Shinyanga women carrying heavy loads. Although most women in the study were approached by a study team member and assessed for eligibility, we also accepted some eligible women who approached a study site and expressed interest in participating (approximately 20 participants). If these women were more likely to participate in the study because they were experiencing or had experienced musculoskeletal pain or disability, our results may be biased away from the null. We believe that the possibility for this bias was low, however, as we were not offering medical advice and/or services as a part of study participation. Although typical for similar studies conducted in comparable populations, self-reported data always represent a potential source of bias or misclassification [6,7]. Indeed, because physical maneuvers or examinations were not possible, we relied on subjective, self-reported scale data. The perception of pain varies: a recent ethnographic study conducted in Botswana highlighted the challenges and complexities behind understanding how individuals perceive and express musculoskeletal pain and disability, particularly in cross-cultural settings, and emphasized the need to consider the social, cultural, and behavioral contexts of the population under investigation [21]. Such differences in communication and context should be considered with the results from this study conducted in a rural part of northern Tanzania. It is for the reasons stated above that we recommend that future studies include a physical exam to increase the specificity of the outcome. Finally, because we carried out multiple statistical tests there was the possibility of type I error; thus, results of this analysis should be interpreted with caution and viewed as exploratory and hypothesis-generating.

Given its predominance in many societies across the globe, low back pain has been the sole focus of much previous MSD research, although research in LMIC remains scant [15]. Findings from such studies include a 35.6% one-year prevalence of reported LBP amongst female farmworkers in rural Nigeria and 61% one-year prevalence reported in female rice farmers living in a rural community in Thailand [22,23]. A systematic review from 2007 found the one-year prevalence of LBP reported amongst adults in various African countries to range anywhere from 14 to 72%; however, these data were not representative of rural populations [11]. Results from the present study, with approximately half of our study participants experiencing LBP over the course of the past year, are comparable to what has been reported in these similar settings and populations. The apparent lack of literature investigating MSDs other than LBP in LMIC make it difficult to make similar comparisons across settings for other assessed body regions. As such, ours may be one of the first studies to attempt to quantify the prevalence of multiple types of musculoskeletal pain (i.e., neck, head, knees, and feet/ankle pain) and related disability amongst women living in a LMIC. Similar to reported LBP, we found a high prevalence of musculoskeletal pain in all assessed body regions: one-year prevalence of pain ranged from 43–61% across assessed body regions, with neck pain within the last 12 months being the most prevalent, although not the most strongly associated with load carrying in this study. We also found a high prevalence of associated disability. Taking also into consideration the high severity of the reported pain, results from our assessment of MSDs reflect a significant amount of disability in this population of Tanzanian women.

Our primary exposure assessment revealed that participants completed an average of 20 load-carrying trips per week while transporting loads weighing an average of 18.8 kg, with typical trips lasting an average of 29.5 minutes. Water, wood, and agricultural products were reported as the most frequently carried items, and, in general, participants reported all types of loads as requiring a great deal of physical exertion to carry. These exposure data reflect the substantial amount of time and physical work required to obtain basic daily household necessities, such as water, food, and fuel, in this population. To put the immense workload of African women into perspective, research using psychophysical methods to provide an estimate of the percent of the female population who could carry various weights for different distances and frequencies during a work shift can be referenced. Although a direct comparison of a Tanzanian women’s workload to a typical American female is difficult, based on the average water load (18.9 kilograms), duration (18.8 minutes) and frequency (4 times per day and 5.3 days/week), and using an average comfortable walking speed of 141.5 cm/second the psychophysical tables indicate that less than 10% of the US female population would find this task acceptable [24,25]. Given this level of exposure, the innovation and implementation of culturally appropriate interventions could ease a significant daily burden of load carrying in such populations. In this region of the world where household welfare greatly depends on females’ capacity to complete daily work, such potential interventions could have a profound impact on the livelihoods of these women and those who depend on them.

Some existing interventions have focused on facilitating water carrying in similar resource-constrained settings. Examples include rolling water wheels such as the Hippo Roller, greater access to bicycles for quicker transport of water to and from homes, and funding to increase the construction of water wells closer to villages [26]. However, our findings indicate that participants in this study population perceived loads *other* than water as requiring greater amounts of exertion to transport: building materials such as rocks, sand, and bricks received the highest exertion scores, and, of the most commonly carried items in this population, wood was rated as requiring the most exertion. This finding may be the result of several factors, including the relatively long average amount of time required to collect wood, or, based on our measurements, its heavy average weight compared to other typically carried loads. This agrees with evidence from other similar populations indicating that firewood was perceived and recorded as the heaviest type of load carried [3]. It could also be likely that the unwieldy size and shape of firewood bundles compared to buckets of water or bags of food make them more difficult to transport. Overall, these findings suggest that while it is imperative to continue to improve water accessibility and the ease of its transport in rural locations, expanding existing interventions to improve the transport of other types of materials could be crucial for increasing access to basic goods, decreasing overall daily workload, and potentially lessening the risk of musculoskeletal injury. However, while the associations we found between LBP and exposure to load carrying provide justification for interventions that focus on these relationships, it is also necessary to establish a stronger evidence base for the impacts of heavy load carrying, including by using physical examinations and other objective measurement methods, particularly amongst women and girls.

Overall, data from the present study highlight the need for further research investigating load-carrying risk factors for MSDs and the impact that related disability has on health and welfare amongst similar populations. If relationships between load carrying and MSDs are confirmed, it will provide even stronger evidence for the development of appropriate interventions or preventive measures to reduce the considerable amount of pain experienced amongst women who carry heavy loads, particularly for the lower back. Because heavy load carrying by women in LMICs is so prevalent, in addressing this largely neglected topic significant strides may be made towards improving the health and wellbeing of women around the world.

## Additional File

The additional file for this article can be found as follows:

10.5334/aogh.2470.s1 Load Carrying and Musculoskeletal Pain Questionnaire. Baseline Survey Load Carrying.

## References

[1] Global Burden of Disease Study C. Global, regional, and national incidence, prevalence, and years lived with disability for 310 diseases and injuries, 1990–2015: A systematic analysis for the Global Burden of Disease Study 2015. Lancet. 2016; 388(10053): 1545–1602. DOI: 10.1016/S0140-6736(16)31678-6 27733282 PMC5055577

[2] Global Burden of Disease Study C. Global, regional, and national incidence, prevalence, and years lived with disability for 301 acute and chronic diseases and injuries in 188 countries, 1990–2013: A systematic analysis for the Global Burden of Disease Study 2013. Lancet. 2015; 386(9995): 743–800. DOI: 10.1016/S0140-6736(15)60692-4 26063472 PMC4561509

[3] Porter G, Hampshire K, Dunn C, et al. Health impacts of pedestrian head-loading: A review of the evidence with particular reference to women and children in sub-Saharan Africa. Soc Sci Med. 2013; 88: 90–97. DOI: 10.1016/j.socscimed.2013.04.010 23702214

[4] Subba S. Women, Woodfuel, and Health in Adamtar Village, Nepal. Gender, Technology, and Development. 1999; 3(3): 361–377. DOI: 10.1080/09718524.1999.11909939

[5] Earth B, Sthapit, S. Uterine Prolapse in rural Nepal: Gender and human rights implications. A mandate for development. Cult Health Sex. 2002; 4(3): 281–296. DOI: 10.1080/13691050110090248

[6] Geere JA, Hunter PR, Jagals P. Domestic water carrying and its implications for health: a review and mixed methods pilot study in Limpopo Province, South Africa. Environ Health. 2010; 9: 52 DOI: 10.1186/1476-069X-9-52 20796292 PMC2939590

[7] Geere JA, Bartram J, Bates L, et al. Carrying water may be a major contributor to disability from musculoskeletal disorders in low income countries: A cross-sectional survey in South Africa, Ghana and Vietnam. J Glob Health. 2018; 8(1): 010406 DOI: 10.7189/jogh.08.010406 29497503 PMC5825974

[8] Lloyd R, Parr B, Davies S, Cooke C. Subjective perceptions of load carriage on the head and back in Xhosa women. Appl Ergon. 2010; 41(4): 522–529. DOI: 10.1016/j.apergo.2009.11.001 19926071

[9] Carroll LJ, Hogg-Johnson S, van der Velde G, et al. Course and prognostic factors for neck pain in the general population: results of the Bone and Joint Decade 2000–2010 Task Force on Neck Pain and Its Associated Disorders. J Manipulative Physiol Ther. 2009; 32(2 Suppl): S87–96. 19251079 10.1016/j.jmpt.2008.11.013

[10] Schierhout GH, Meyers JE, Bridger RS. Work related musculoskeletal disorders and ergonomic stressors in the South African workforce. Occup Environ Med. 1995; 52(1): 46–50. DOI: 10.1136/oem.52.1.46 7697140 PMC1128149

[11] Louw QA, Morris LD, Grimmer-Somers K. The prevalence of low back pain in Africa: a systematic review. BMC Musculoskelet Disord. 2007; 8: 105 DOI: 10.1186/1471-2474-8-105 17976240 PMC2198912

[12] van Vuuren BJ, Becker PJ, van Heerden HJ, Zinzen E, Meeusen R. Lower back problems and occupational risk factors in a South African steel industry. Am J Ind Med. 2005; 47(5): 451–457. DOI: 10.1002/ajim.20164 15828071

[13] Borg GA. Psychophysical bases of perceived exertion. Med Sci Sports Exerc. 1982; 14(5): 377–381. DOI: 10.1249/00005768-198205000-00012 7154893

[14] Krebs EE, Carey TS, Weinberger M. Accuracy of the pain numeric rating scale as a screening test in primary care. J Gen Intern Med. 2007; 22(10): 1453–1458. DOI: 10.1007/s11606-007-0321-2 17668269 PMC2305860

[15] Ogunbode AM, Adebusoye LA, Alonge TO. Prevalence of low back pain and associated risk factors amongst adult patients presenting to a Nigerian family practice clinic, a hospital-based study. Afr J Prim Health Care Fam Med. 2013; 5(1). DOI: 10.4102/phcfm.v5i1.441

[16] MacLellan GA, Dunlevy C, O’Malley E, et al. Musculoskeletal pain profile of obese individuals attending a multidisciplinary weight management service. Pain. 2017; 158(7): 1342–1353. DOI: 10.1097/j.pain.0000000000000918 28383311

[17] Peltonen M, Lindroos AK, Torgerson JS. Musculoskeletal pain in the obese: a comparison with a general population and long-term changes after conventional and surgical obesity treatment. Pain. 2003; 104(3): 549–557. DOI: 10.1016/S0304-3959(03)00091-5 12927627

[18] Mogren IM. BMI, pain and hyper-mobility are determinants of long-term outcome for women with low back pain and pelvic pain during pregnancy. Eur Spine J. 2006; 15(7): 1093–1102. DOI: 10.1007/s00586-005-0004-9 16404613 PMC3233935

[19] Mannion CA, Vinturache AE, McDonald SW, Tough SC. The Influence of Back Pain and Urinary Incontinence on Daily Tasks of Mothers at 12 Months Postpartum. PLoS One. 2015; 10(6): e0129615 DOI: 10.1371/journal.pone.0129615 26083252 PMC4471341

[20] Bliddal M, Pottegard A, Kirkegaard H, et al. Association of Pre-Pregnancy Body Mass Index, Pregnancy-Related Weight Changes, and Parity With the Risk of Developing Degenerative Musculoskeletal Conditions. Arthritis Rheumatol. 2016; 68(5): 1156–1164. 26714126 10.1002/art.39565

[21] Hondras M, Hartvigsen J, Myburgh C, Johannessen H. Everyday burden of musculoskeletal conditions among villagers in rural Botswana: A focused ethnography. J Rehabil Med. 2016; 48(5): 449–455. DOI: 10.2340/16501977-2083 27058751

[22] Omokhodion FO. Low back pain in a rural community in South West Nigeria. West Afr J Med. 2002; 21(2): 87–90. 12403024

[23] Taechasubamorn P, Nopkesorn T, Pannarunothai S. Prevalence of low back pain among rice farmers in a rural community in Thailand. J Med Assoc Thai. 2011; 94(5): 616–621. 21675453

[24] Bohannon RW. Comfortable and maximum walking speed of adults aged 20–79 years: reference values and determinants. Age Ageing. 1997; 26(1): 15–19. DOI: 10.1093/ageing/26.1.15 9143432

[25] Ciriello VM, Snook SH, Hughes GJ. Further studies of psychophysically determined maximal acceptable weights and forces. Human Factors. 1993; 35(11): 175–186. DOI: 10.1177/001872089303500110 8509102

[26] This innovative water carrier is changing lives in rural Africa Raver/Business Insider G; 2016.

