# Supplementary material for: The Impact of Heavy Load Carrying on Musculoskeletal Pain and Disability Among Women in Shinyanga Region, Tanzania
Source: Ann Glob Health. 2020 Feb 21;86(1):17. doi: 10.5334/aogh.2470 (PMC7034319; doi:10.5334/aogh.2470)
Supplement: Load Carrying and Musculoskeletal Pain Questionnaire. — Baseline Survey Load Carrying. [file agh-86-1-2470-s1.pdf]

Study ID #: ____________________ V1.5

Baseline Survey

Interview date	☐☐-☐☐-☐☐ *(dd-mm-yy)*
Location of interview	
Data collector name	
Time interview started	*(24hr clock)* ☐☐:☐☐

Date of entry into study database (dd-mm-yy): ☐☐-☐☐-☐☐ Initials: ☐☐

A. Demographics

**Introduction 1**	**READ ALOUD:** *Thank you for talking with me today. Today, we're going to complete a survey for the study. I'll ask you questions about you and your everyday life. We'll talk about loads that you carry, health, and other related questions. Your responses will remain entirely confidential, so please keep this in mind and please answer these questions as honestly as possible. If you have any questions or if anything I ask is unclear, please tell me and I'll go back over the question.*<br><br>*I'd like to start by asking you a few questions about yourself and members of your household.*

	QUESTION	RESPONSE CODE	FILL IN RESPONSE
A1	In what month and year were YOU born?	99. Don't know month<br>9999. Don't know year	☐☐ mm<br>-<br>☐☐☐☐ yy
A2	How old were you at your last birthday?	Age in completed years	☐☐
A3	What language do you primarily speak at home?	1. Swahili<br>2. English<br>3. Sukuma<br>4. Other (**specify**): ____________	☐
A4	Are you currently enrolled in school?	1. No → **Skip to A6**<br>2. Yes	☐
A5	How many days per week do you go to school in a typical school week?		☐
A6	What is your current marital status?	1. Single/never married/no partner →**Skip to A8**<br>2. Married (only wife)<br>3. Married (one of several wives)<br>4. Unmarried, with partner<br>5. Widowed<br>6. Divorced<br>7. Separated	☐

A7	Do you currently live with a partner?	1. No<br>2. Yes	☐
A8	What is your primary occupation? *[check all that apply]*	☐ Farmer or farm worker<br>☐ Student<br>☐ Business<br>☐ Housewife<br>☐ Shopkeeper<br>☐ Unemployed<br>☐ Other (specify): ____________________	
A9	Are you currently working? That includes working to maintain your home or farm.	1. No → **Skip to A11**<br>2. Yes	☐
A10	How many days of work do you have in a typical week?		☐
A11	Are you head of household?	1. No<br>2. Yes, head of household or married to head of household	☐
A12	Including yourself, how many people live in your household and regularly eat meals together? By regularly, I mean at least 4 times a week.		☐☐<br>people

B. Load carrying

**Introduction 2**	**READ ALOUD:** *Now I am going to ask you some questions regarding the loads that you typically carry. What I mean by "load" is any time you carried water, wood, coal, charcoal, food, or other things that women typically carry in Shinyanga.*

	QUESTION	RESPONSE CODE	FILL IN RESPONSE

B1	At what age did you begin carrying loads either on your head, on your back, on your shoulder, or in front of you?	Age in years	☐☐
B2	In the last 7 days, how many days did you carry loads?	Number in days	☐
B3	In the last 7 days, on the days you carried loads, how many times per day did you carry loads?	Number of loads per day	☐☐
B4	In the last 7 days, on average, how many minutes did it take you to carry a single load?	Time in minutes	☐☐☐
B4.1	In the last 7 days, how many minutes was your shortest trip carrying a load?	Time in minutes	☐☐☐
B4.2	In the last 7 days, how many minutes was your longest trip carrying a load?	Time in minutes	☐☐☐
B5	In a normal week, are you the only person who carries loads in the household?	1. No<br>2. Yes → **Skip to B7**	☐
B5.1	If not, who else carries loads?<br><br>*[check all that apply]*	☐ Husband<br>☐ Son<br>☐ Daughter<br>☐ Mother<br>☐ Father<br>☐ Sister<br>☐ Sister-in-law<br>☐ Brother<br>☐ Other (specify here →)	

B5.2	Are any of these people under the age of 15?	1. No<br>2. Yes (specify person and age →)	
B6	Have you ever carried any of the following loads?<br>*[check all mentioned]*	☐ Water<br>☐ Wood<br>☐ Coal or charcoal<br>☐ Agricultural products (e.g., maize, crops, fruits, etc.)<br>☐ Sand<br>☐ Rocks<br>☐ Other (specify here → and indicate on B7, B8 and B9)	

			**Item not carried**	**On how many days in the last seven days did you carry this item?**	**In the last seven days, approximately how many times per day did you carry this item?**	**In the last seven days, approximately how many minutes was the typical trip carrying this item?**
B7	In the last seven days, have you carried:	**Water**	☐	☐	☐☐	☐☐☐
		**Wood**	☐	☐	☐☐	☐☐☐
		**Sand**	☐	☐	☐☐	☐☐☐
		**Rocks**	☐	☐	☐☐	☐☐☐
		**Food/Crops**	☐	☐	☐☐	☐☐☐

		Coal/Charcoal	☐	☐	☐☐	☐☐☐
		Other items (from B6)				

*** Cross check with B2 and B4.2**

			Item not carried	BORG rating
B8	*(Show participant printed BORG scale)* Think about the last time you carried the following items. On this scale, where 0 is not hard and 10 is very, very hard, what number would you pick to describe how difficult it feels to carry the following items?	**Water**	☐	☐☐
		**Wood**	☐	☐☐
		**Sand**	☐	☐☐
		**Rocks**	☐	☐☐
		**Food/Crops**	☐	☐☐
		**Coal/Charcoal**	☐	☐☐

		**Other items (from B6)**		

			Item not carried	Less difficult	More difficult	It feels the same
B9	Think about the last time you carried a 20L bucket full of water. Based off of how you feel today, is it more or less difficult to carry the following items, compared to a bucket full of water: *(Check one choice for each load category):*	**Wood (Show participant bundle of wood)**	☐	☐	☐	☐
		**Sand**	☐	☐	☐	☐
		**Rocks**	☐	☐	☐	☐
		**Flour (Show participant bag full of flour)**	☐	☐	☐	☐
		**Crops (Show participant bag full of potatoes)**				
		**Coal/Charcoal (Show participant bag full of coal)**	☐	☐	☐	☐
		**Other items (from B6)**				

B10	Based on your most frequent route, is your walk:<br>*[check all that apply]*	☐ Flat<br>☐ Flat with small hills<br>☐ Flat with some larger hills<br>☐ Hilly	
B11	When you are carrying loads, on what sort of paths do you walk on? *[check all that apply]*	☐ A dirt road also used for cars<br>☐ A dirt road only used for people and animals<br>☐ A paved road<br>☐ A rocky path<br>☐ A sandy path<br>☐ A grassy path<br>☐ Other (specify) →	

C. Pain Assessment

**Introduction 3**	**READ ALOUD:** *Now I am going to ask you some questions about your health in order to gain a better understanding of how it might be related to load carrying.*

NECK

			In the last 12 months?	In the last month?
C1	Have you had pain in your neck lasting more than 3 days:	1. No<br>2. Yes	☐<br>**If 1 → Skip to C8 and mark "No Pain" in C36**	☐<br>**If 1 → Skip to C4**
C2	How many days in the past month did you have pain in your neck?	Number in days →	☐☐	
C3	How many days of work or school did you have to miss in the past 30 days because of the neck pain, if any?	Number in days →	☐☐	

C4	Using the scale on this card *[give them the showcard for part C],* in the last 12 months, how would you rate the pain in your neck at its worst?		☐☐

		**Cooking**	**Caring for the home**	**Caring for others (children, or older people)**	**Collecting wood, water, or other household necessities**	**Other (specify):**
C5	In the last 12 months, did the neck pain prevent you from doing any of the following activities? I'm going to read a list and you can tell me if you were prevented from….	☐	☐	☐	☐	

C6	In the last 12 months, what sort of problems have you had with your neck?<br>*[check all that apply]*	☐ Pain<br>☐ Stiffness<br>☐ Spasm<br>☐ Unable to move your head<br>☐ Burning, numbness or tingling<br>☐ Other (specify →)	
C6.1	Does the neck pain or discomfort go down your arm?	1. No<br>2. Yes	☐
C7	Is there anything that you think may have caused or contributed to this problem?	Record response here →	

HEAD

			**In the last 12 months?**	**In the last month?**
C8	Different from when you might have had malaria, or any other fever, have you had head problems lasting more than 3 days:	1. No<br>2. Yes	☐<br>**If 1 → Skip to C15 and mark "No Pain" in C36**	☐<br>**If 1 → Skip to C11**

C9	How many days in the past month did you have pain in your head?	Number in days →	☐☐
C10	How many days of work or school did you have to miss in the past 30 days because of the head pain, if any?	Number in days →	☐☐
C11	Using the scale on this card *[give them the showcard for part C]*, in the last 12 months, how would you rate the pain in your head at its worst?		☐☐

		**Cooking**	**Caring for the home**	**Caring for others (children, or older people)**	**Collecting wood, water, or other household necessities**	**Other (specify):**
C12	In the last 12 months, did the head pain prevent you from doing any of the following activities? I'm going to read a list and you can tell me if you were prevented from….	☐	☐	☐	☐	

C13	In the last 12 months, what sort of problems have you had with your head?<br>*[check all that apply]*	☐ Pain<br>☐ Stiffness<br>☐ Spasm<br>☐ Unable to move your head<br>☐ Burning, numbness or tingling<br>☐ Other (record response here →)	

C14	Is there anything that you think may have caused or contributed to this problem?	Record response here →	

BACK

			In the last 12 months?	In the last month?
C15	Have you had back problems lasting more than 3 days:	1. No<br>2. Yes	☐<br>**If 1 → Skip to C22 and mark "No Pain" in C36**	☐<br>**If 1 → Skip to C18**
C16	How many days in the past month did you have pain in your back?	Number in days →	☐☐	
C17	How many days of work or school did you have to miss in the past 30 days because of the back pain, if any?	Number in days →	☐☐	
C18	Using the scale on this card *[give them the showcard for part C]*, in the last 12 months, how would you rate the pain in your back at its worst?		☐☐	

		Cooking	Caring for the home	Caring for others (children, or older people)	Collecting wood, water, or other household necessities	Other (specify):
C19	In the last 12 months, did the back pain prevent you from doing any of the following activities? I'm going to read a list and you can tell me if you were prevented from….	☐	☐	☐	☐	

C20	In the last 12 months, what sort of problems have you had with your back?<br>*[check all that apply]*	☐ Pain<br>☐ Stiffness<br>☐ Spasm<br>☐ Burning, numbness or tingling<br>☐ Other (specify →)	
C20.1	Does the back pain or discomfort go down your leg?	1. No<br>2. Yes	☐
C21	Is there anything that you think may have caused or contributed to this problem?	Record response here →	

KNEES

			**In the last 12 months?**	**In the last month?**
C22	Have you had knee problems lasting more than 3 days:	1. No<br>2. Yes	☐<br>**If 1 → Skip to C29 and mark "No Pain" in C36**	☐<br>**If 1 → Skip to C25**
C23	How many days in the past month did you have pain in your knees?	Number in days →	☐☐	
C24	How many days of work or school did you have to miss in the past 30 days because of the knee pain, if any?	Number in days →	☐☐	
C25	Using the scale on this card *[give them the showcard for part C]*, in the last 12 months, how would you rate the pain in your knees at its worst?		☐☐	

C26	In the last 12 months, did the knee pain prevent you from	**Cooking**	**Caring for the home**	**Caring for**	**Collecting wood, water,**	**Other (specify):**

	doing any of the following activities? I'm going to read a list and you can tell me if you were prevented from….			**others (children, or older people)**	**or other household necessities**	
		☐	☐	☐	☐	

C27	In the last 12 months, what sort of problems have you had with your knees?<br>*[check all that apply]*	☐ Pain<br>☐ Stiffness<br>☐ Swelling<br>☐ Other (specify →)	
C28	Is there anything that you think may have caused or contributed to this problem?	Record response here →	

FOOT/ANKLE(S)

			**In the last 12 months?**	**In the last month?**
C29	Have you had foot/ankle problems lasting more than 3 days:	1. No<br>2. Yes	☐<br>**If 1 → Skip to C36 and mark "No Pain" in C36**	☐<br>**If 1 → Skip to C32**
C30	How many days in the past month did you have pain in your foot/ankle(s)?	Number in days →	☐☐	
C31	How many days of work or school did you have to miss in the past 30 days because of the foot/ankle pain, if any?	Number in days →	☐☐	
C32	Using the scale on this card *[give them the showcard for part C]*, in the last 12 months, how would you rate the pain in your feet/ankle(s) at its worst?		☐☐	

C33	In the last 12 months, did the feet/ankle pain prevent you from doing any of the following activities? I'm going to read a list and you can tell me if you were prevented from….	**Cooking**	**Caring for the home**	**Caring for others (children, or older people)**	**Collecting wood, water, or other household necessities**	**Other (specify):**
		☐	☐	☐	☐	

C34	In the last 12 months, what sort of problems have you had with your foot/ankle(s)?<br>*[check all that apply]*	☐ Pain<br>☐ Stiffness<br>☐ Swelling<br>☐ Other (specify →)	
C35	Is there anything that you think may have caused or contributed to this problem?	Record response here →	

C36	In the past year, have you seen a health or medical care provider for problem(s) with your:		**No Pain**	**Have not seen a healthcare provider**	**If yes, how many times in the past year have you seen a healthcare provider?**	**Was the healthcare provider able to help? (was there any improvement in pain, even without complete resolution)?**
		**Neck**	☐	☐	☐☐☐	☐
		**Head**	☐	☐	☐☐☐	☐
		**Back**	☐	☐	☐☐☐	☐
		**Knee(s)**	☐	☐	☐☐☐	☐

		**Foot/Ankle**	☐	☐	☐☐☐	☐

C37	Is there any other pain you are experiencing that you think is relevant that you'd like to tell me about?	Record response here →	

D. Overall/Reproductive Health

**Introduction 4**	**READ ALOUD:** *Now I'd like to ask a few questions about your overall health and how you feel, and some reproductive health questions. Some of these questions are very private, but your information will be kept confidential, and no one can hear the answers you give me today.*		
	QUESTION	RESPONSE CODE	FILL IN RESPONSE
D1	How would you describe your overall health? We'll use a scale of 1 – 5, [1 being Poor and 5 being Excellent]	1. Poor<br>2. Fair<br>3. Good<br>4. Very Good<br>5. Excellent	☐
D2	Do you have any children?	1. No → **Skip to D4**<br>2. Yes	☐
D2.1	If yes, how many?		☐☐
D3	At what age did you have your first child?		☐☐
D4	How many times have you been pregnant?		☐☐
D5	How many times have you delivered a baby?		☐☐

D6	Are you currently pregnant?	1. No<br>2. Yes	☐
D7	Do you ever have pain while urinating?	1. No → **Skip to D8**<br>2. Yes	☐
D7.1	If yes, how many times in the last 30 days did you have pain while urinating?		☐☐
D8	Are you currently sexually active?	1. No → **Skip to D9**<br>2. Yes	☐
D8.1	Do you ever have pain while having intercourse?	1. No → **Skip to D9**<br>2. Yes	☐
D8.2	If yes, how many times in the last 30 days did you have pain while having intercourse?		☐☐
D9	Do you have any problems holding your urine?	1. No → **Skip to D10**<br>2. Yes	☐
D9.1	If yes, how often do you experience problems holding in your urine?	1. All the time<br>2. Most of the time<br>3. Some of the time<br>4. Rarely<br>5. Never	☐
D10	Except for the normal cramps and pressure you might feel during your monthly period, have you ever experienced a feeling of pressure in your lower abdomen?	1. No → **Skip to D11**<br>2. Yes	☐
D10.1	If yes, how often does this pressure bother you?	1. All the time<br>2. Most of the time<br>3. Some of the time<br>4. Rarely<br>5. Never	☐

D10.2	How much does this pressure bother you?	1. A great deal<br>2. A lot<br>3. A moderate amount<br>4. A little<br>5. Not at all	☐
D10.3	Can you please explain/elaborate on the pressure that you are experiencing?	Record response here →	
D11	Have you ever experienced a feeling of heaviness or dullness in the pelvic area?	1. No → **Skip to D12**<br>2. Yes	☐
D11.1	If yes, how often does this feeling bother you?	1. All the time<br>2. Most of the time<br>3. Some of the time<br>4. Rarely<br>5. Never	☐
D11.2	How much does this feeling bother you?	1. A great deal<br>2. A lot<br>3. A moderate amount<br>4. A little<br>5. Not at all	☐
D12	Have you ever noticed a bulge or something falling out of your vaginal area that you can feel? I am not referring to menstrual blood or clots, which are normal.	1. No → **Skip to part E**<br>2. Yes	☐
D12.1	Did it ever stop you from working or keep you home?	1. No<br>2. Yes	☐
D12.2	How often does this feeling bother you?	1. All the time<br>2. Most of the time<br>3. Some of the time<br>4. Rarely<br>5. Never	☐
D12.3	How much does this feeling bother you?	1. A great deal<br>2. A lot<br>3. A moderate amount<br>4. A little<br>5. Not at all	☐

D12.4	Does the bulge return back inside of you?	1. No<br>2. Yes	☐
D13	Do you have a history of C-section or any other gynecological surgery?	1. No<br>2. Yes	☐

**E. Weighing/Assessment of Load**	
**Introduction 5**	**READ ALOUD:** *I'd like to visually assess the load that you have with you today and take a couple of notes. I would also like to weigh and measure you and the load that you are carrying. (NOTE: The questions in this section are to be completed by the study team member.)*

E1	Weigh load by placing on the scale.	Record weight of load here in lbs →	☐☐ ☐☐
E2	What are the contents of the load?	Note contents of load here →	
E3	In what is the load being carried?	☐ Basket<br>☐ Bucket<br>☐ Container with a lid<br>☐ Sack/Bag<br>☐ Other (specify →)	
E4	How does the participant carry the load?	☐ On head<br>☐ On back<br>☐ On shoulder<br>☐ In front of body<br>☐ Other (specify →)	
E5	Is the participant carrying anything else?	1. No → skip to E6<br>2. Yes	☐

E5.1	If yes, what is she carrying? *[check all that apply]*	☐ Water<br>☐ Wood<br>☐ Coal/Charcoal<br>☐ Agricultural products (e.g., maize, crops, fruits, etc.)<br>☐ Gravel or sand<br>☐ Baby<br>☐ Other (specify) →	
E5.2	If yes, how is she carrying it?	☐ On back<br>☐ On shoulder<br>☐ In front of body<br>☐ In hand<br>☐ Other (specify) →	
E5.3	If yes, what is she carrying it in?	☐ Basket<br>☐ Bucket<br>☐ Container with lid<br>☐ Sack/Bag<br>☐ Other (specify) →	
E6	What sort of footwear is the participant wearing?	☐ Sandals<br>☐ Closed-toed shoes<br>☐ No shoes<br>☐ Other (specify) →	
E7	Have the participant step on the scale and record her weight.	Record participant weight (kg) here →	☐☐☐.☐
E8	Measure the participant's height.	Record participant height (cm) here →	☐☐☐.☐

END OF SURVEY

Conclusion.

READ ALOUD: That is all we need to ask you today. Is there anything else you want to tell us about?
Thank you for your participation.

Time interview ended	*(24hr clock)* ☐☐:☐☐	
OBSERVATIONS ON THE INTERVIEW		
**INSTRUCTIONS**: **Note any unusual circumstances (i.e. interruptions, concerns about response validity) that would be necessary for supervisors and the analysis of this questionnaire. If more space is needed, use the additional notes section on the last page of the survey.**		
